# Calpain/calpstatin pathway in COVID‐19: Role of dantrolene

**DOI:** 10.1111/jcmm.18005

**Published:** 2023-10-18

**Authors:** Hayder M. Al‐Kuraishy, Ali I. Al‐Gareeb, Maisra M. El‐Bouseary, Athanasios Alexiou, Marios Papadakis, Gaber El‐Saber Batiha

**Affiliations:** ^1^ Department of Clinical pharmacology and Medicine, College of Medicine Al‐Mustansiriya University Baghdad Iraq; ^2^ Department of Pharmaceutical Microbiology, Faculty of Pharmacy Tanta University Tanta Egypt; ^3^ Department of Science and Engineering Novel Global Community Educational Foundation Hebersham New South Wales Australia; ^4^ AFNP Med Wien Austria; ^5^ Department of Surgery II University Hospital Witten‐Herdecke, University of Witten‐Herdecke Wuppertal Germany; ^6^ Department of Pharmacology and Therapeutics, Faculty of Veterinary Medicine Damanhour University Damanhour Egypt

Dear Editor,

The current coronavirus disease 2019 (COVID‐19) pandemic, which affects the world in a mysterious way, leads to high morbidity and mortality exceeding a half billion.^1^ COVID‐19 is regarded as a respiratory viral disease that is asymptomatic in the majority of cases.^1^ However, in severe cases, it may lead to acute lung injury (ALI) and acute respiratory distress syndrome (ARDS).^2^ COVID‐19 is caused by a novel virus called SARS‐CoV‐2, which exploits angiotensin converting enzyme 2 (ACE2) and other non‐specific receptors as an entry point for target cells.^2^

SARS‐CoV‐2 infection at the cellular level can induce endoplasmic reticulum (ER) stress and mitochondrial dysfunction, leading to Ca^2+^ release and subsequent activation of inflammatory signalling pathways.^3^ SARS‐CoV‐2 protein E has the ability to form a specific channel in the ER membrane, leading to an increase in the release of Ca^2+^ from the ER and the development of an intracellular Ca^2+^ load.^3^ Therefore, Ca^2+^‐channel blockers could be effective in the management of COVID‐19. A preclinical study demonstrated that Ca^2+^‐channel blocker amlodipine inhibits SARS‐CoV‐2 proliferation in cell culture.^11^ Increasing intracellular Ca^2+^ promotes expression of the calpain/calpastatin system (CCS), which includes a series of proteolytic proteases known as calpains and their inhibitor, calpastatin.^4^ Calpain is an intracellular protease that catalyses proteolysis and is involved in the regulation of intracellular Ca^2+^ which is involved in autophagy, cell proliferation, apoptosis, and cell deaths.^4^ Calpain consists of a large catalytic subunit and a small regulatory subunit. Calpain is highly expressed in most tissues and is a so‐called ubiquitous protease involved in the degradation of various substrates like amyloid precursor protein.^4^ Calpastatin is an endogenous inhibitor of calpain and prevents calpain‐mediated activation of nuclear factor kappa B (NF‐κB). Of note, intracellular Ca^2+^ triggers activation of calpain, leading to a series of proteolytic actions and the development of hyperinflammation and tissue injury.^4^

It has been shown that downregulation of ACE2 by SARS‐CoV‐2 infection promotes increment of pro‐inflammatory angiotensin II (AngII) and reduces expression of anti‐inflammatory Ang1–7.^2^ Besides, AngII activates expression of calpain, causing endothelial dysfunction,^5^ a hallmark of COVID‐19 pathology and associated critical complications. In addition, the exaggerated immune response in severe COVID‐19 induces expression of inflammatory signalling pathways like NF‐κB, NLRP3 inflammasome, STAT3, and p38MAPK. Due to these immunological events, pro‐inflammatory cytokines are highly activated, leading to hypercytokinemia.^6^ Remarkably, calpain enhances entry of SARS‐CoV‐2 by increasing expression of voltage‐gated Ca^2+^ channels and endocytosis.^7^ As well, activated calpain and inhibited calpastatin by SARS‐CoV‐2 trigger the development of a cytokine storm,^7^ which is linked with COVID‐19 severity. These observations suggest a crosstalk between calpain and the immunoinflammatory response in COVID‐19. Therefore, modulation of CCS through activation of calpastatin and inhibition of calpain could be effective in the management of COVID‐19.

In this bargain, modulation of intracellular Ca^2+^ during SARS‐CoV‐2 infection can reduce deregulation of CCS. Dantrolene is a ryanodine receptor antagonist that blocks the release of Ca^2+^ from the sarcoplasmic reticulum and is used in the management of malignant hyperthermia.^3^ A review conducted by Al‐Kazmi et al.^3^ illustrated that dantrolene could be effective in the management of COVID‐19 by different mechanisms, including enhancement of the clearance of SARS‐CoV‐2, inhibition of SARS‐CoV‐2‐induced endothelial dysfunction and coagulopathy, and attenuation of the release of pro‐inflammatory cytokines. Besides, dantrolene has the ability to inhibit expression of calpain‐1 and increase expression of calpastatin in experimental cancer cachexia.^8^ In early COVID‐19, dantrolene was repurposed as a high potential in treating COVID‐19 patients and reducing its morbidity and mortality. Dantrolene was expected to ameliorate the detrimental effects of SARS‐CoV‐2 in host cells.^12^

In a similar way, boceprevir like other calpain inhibitor inhibits SARS‐CoV‐2 replication through inhibition of the viral main protease in cell cultures.^9^ Moreover, calpain inhibitors II and XII, and GC376 analogues, which similarly inhibit both calpain/cathepsin and the viral main protease, they reduce SARS‐CoV‐2 proliferation and entry to the target cells through L‐cathepsin.^10^

Taken together, modulation of CCS by dantrolene could be effective in the management of COVID‐19 by inhibiting the release of pro‐inflammatory cytokines and coagulopathy. This letter prompts a clinical trial to confirm the potential effectiveness of dantrolene in COVID‐19 management regarding calpain/calpstatin pathway.

## AUTHOR CONTRIBUTIONS

**Hayder M. Al‐kuraishy:** Conceptualization (equal); data curation (equal); writing – original draft (equal). **Ali I. Al‐Gareeb:** Conceptualization (equal); data curation (equal); writing – original draft (equal). **Maisra M. El‐Bouseary:** Data curation (equal); writing – review and editing (equal). **Athanasios Alexiou:** Data curation (equal); writing – review and editing (equal). **Marios Papadakis:** Data curation (equal); writing – review and editing (equal). **Gaber El‐Saber Batiha:** Data curation (equal); writing – review and editing (equal).

## FUNDING INFORMATION

No funding was received for conducting this study.

## CONFLICT OF INTEREST STATEMENT

The authors have no competing interests to declare that are relevant to the content of this article.

## Data Availability

All data generated or analysed during this study are included in this published article.
